# Large Language Models for Pediatric Differential Diagnoses in Rural Health Care: Multicenter Retrospective Cohort Study Comparing GPT-3 With Pediatrician Performance

**DOI:** 10.2196/65263

**Published:** 2025-03-19

**Authors:** Masab Mansoor, Andrew F Ibrahim, David Grindem, Asad Baig

**Affiliations:** 1 Edward Via College of Osteopathic Medicine, 4408 Bon Aire Dr, Monroe, LA, 71203, United States, 1 5045213500; 2 Texas Tech University Health Sciences Center School of Medicine, Lubbock, TX, United States; 3 Mayo Clinic, Rochester, MN, United States; 4 Department of Radiology, Columbia University Medical Center, New York, NY, United States

**Keywords:** natural language processing, NLP, machine learning, ML, artificial intelligence, language model, large language model, LLM, generative pretrained transformer, GPT, pediatrics

## Abstract

**Background:**

Rural health care providers face unique challenges such as limited specialist access and high patient volumes, making accurate diagnostic support tools essential. Large language models like GPT-3 have demonstrated potential in clinical decision support but remain understudied in pediatric differential diagnosis.

**Objective:**

This study aims to evaluate the diagnostic accuracy and reliability of a fine-tuned GPT-3 model compared to board-certified pediatricians in rural health care settings.

**Methods:**

This multicenter retrospective cohort study analyzed 500 pediatric encounters (ages 0‐18 years; n=261, 52.2% female) from rural health care organizations in Central Louisiana between January 2020 and December 2021. The GPT-3 model (DaVinci version) was fine-tuned using the OpenAI application programming interface and trained on 350 encounters, with 150 reserved for testing. Five board-certified pediatricians (mean experience: 12, SD 5.8 years) provided reference standard diagnoses. Model performance was assessed using accuracy, sensitivity, specificity, and subgroup analyses.

**Results:**

The GPT-3 model achieved an accuracy of 87.3% (131/150 cases), sensitivity of 85% (95% CI 82%‐88%), and specificity of 90% (95% CI 87%‐93%), comparable to pediatricians’ accuracy of 91.3% (137/150 cases; *P*=.47). Performance was consistent across age groups (0‐5 years: 54/62, 87%; 6‐12 years: 47/53, 89%; 13‐18 years: 30/35, 86%) and common complaints (fever: 36/39, 92%; abdominal pain: 20/23, 87%). For rare diagnoses (n=20), accuracy was slightly lower (16/20, 80%) but comparable to pediatricians (17/20, 85%; *P*=.62).

**Conclusions:**

This study demonstrates that a fine-tuned GPT-3 model can provide diagnostic support comparable to pediatricians, particularly for common presentations, in rural health care. Further validation in diverse populations is necessary before clinical implementation.

## Introduction

The rapid advancement of artificial intelligence (AI) has led to the development of large language models (LLMs) that demonstrate sophisticated capabilities in understanding and analyzing human language [1]. Recent studies have shown promising applications of LLMs in health care, particularly in clinical decision support, medical knowledge synthesis, and diagnostic assistance [2-4]. However, their reliability and accuracy in specialized medical domains, especially pediatric care in resource-constrained settings, require thorough evaluation.

Differential diagnosis in pediatrics presents unique challenges that distinguish it from adult medicine. Young patients often cannot articulate their symptoms clearly, presentations can be atypical, and the range of potential diagnoses varies significantly with age. Recent systematic reviews have shown that diagnostic errors occur in “appreciable amounts” of pediatric encounters, with higher rates in rural and underserved areas [5]. These errors can lead to delayed treatment, inappropriate interventions, and potentially adverse outcomes.

The application of LLMs in clinical decision support has shown initial promise. Studies using GPT-3 and similar models have reported accuracies ranging from 75% to 85% in generating differential diagnoses for adult cases [6]. Notably, Steinberg et al [7] demonstrated that LLMs could achieve 82% accuracy in analyzing electronic health record (EHR) data for diagnostic support. However, pediatric applications remain underexplored, with limited studies specifically examining LLM performance in child and adolescent cases.

Rural health care settings face particular challenges that could benefit from LLM-based support tools. These areas often experience physician shortages, with providers managing high patient volumes and limited access to specialist consultation [8]. A survey of rural pediatric practices found that 52% of rural pediatricians report difficulty obtaining timely specialist input for complex cases [9]. Additionally, rural providers often work in isolation, managing a broad spectrum of conditions with fewer diagnostic resources compared to urban centers [10].

Previous evaluations of AI in pediatric diagnosis have largely focused on specific conditions or imaging-based applications rather than broad differential diagnosis. For instance, Wu et al [11] achieved 97.45% accuracy in pediatric otitis media interpretation using deep learning models, while other studies have demonstrated AI’s effectiveness in detecting pediatric pneumonia from chest x-rays or identifying developmental disorders through automated screening tools. However, these models are often constrained by narrow diagnostic scopes, lack interpretability, and are not readily adaptable to general pediatric clinical reasoning.

Recent studies have begun to explore the application of LLMs in pediatric clinical settings. For example, Nian et al [12] found that ChatGPT and Google Gemini performed inadequately in providing recommendations for managing developmental dysplasia of the hip compared to expert guidelines, raising concerns about reliability in pediatric decision-making. Similarly, Wang et al [13] developed an LLM-based framework for pediatric obstructive sleep apnea management, highlighting the potential for specialized fine-tuning to improve diagnostic accuracy in specific pediatric conditions. Miyake et al [14] explored the role of AI-driven LLMs in pediatric surgery, emphasizing challenges related to real-time intraoperative decision support. Furthermore, Raza et al [15] investigated LLM applications in analyzing parental transcripts for children with congenital heart disease, demonstrating their potential role in augmenting thematic analysis in pediatric health care.

Despite these developments, comprehensive evaluations of LLMs in general pediatric differential diagnosis remain scarce. Many existing studies focus on narrow applications, lack real-world clinical validation, or fail to address age-specific nuances in pediatric presentations. Additionally, research on LLM utility in rural settings, where pediatricians may have limited access to specialist support, is particularly lacking. This study aims to bridge these gaps by systematically evaluating LLM performance in general pediatric differential diagnosis, with a focus on rural applicability and real-world clinical decision support.

The emergence of newer LLM architectures and their potential application in health care necessitates rigorous evaluation in real-world clinical settings [16]. While preliminary studies suggest promise, questions remain about their reliability, safety, and integration into clinical workflows [17]. Furthermore, the unique aspects of pediatric care—including age-specific disease presentations, developmental considerations, and the critical nature of early accurate diagnosis—require specific validation of these tools in pediatric populations [18].

This study addresses these knowledge gaps by evaluating the performance of a fine-tuned GPT-3 model in generating pediatric differential diagnoses within rural health care settings. By comparing the model’s performance with that of experienced pediatricians across various age groups and presenting complaints, we aim to assess its potential as a clinical decision support tool. The findings could inform the development of AI-assisted diagnostic tools specifically tailored to the needs of rural pediatric health care providers.

## Methods

### Study Design and Setting

**Figure 1.**
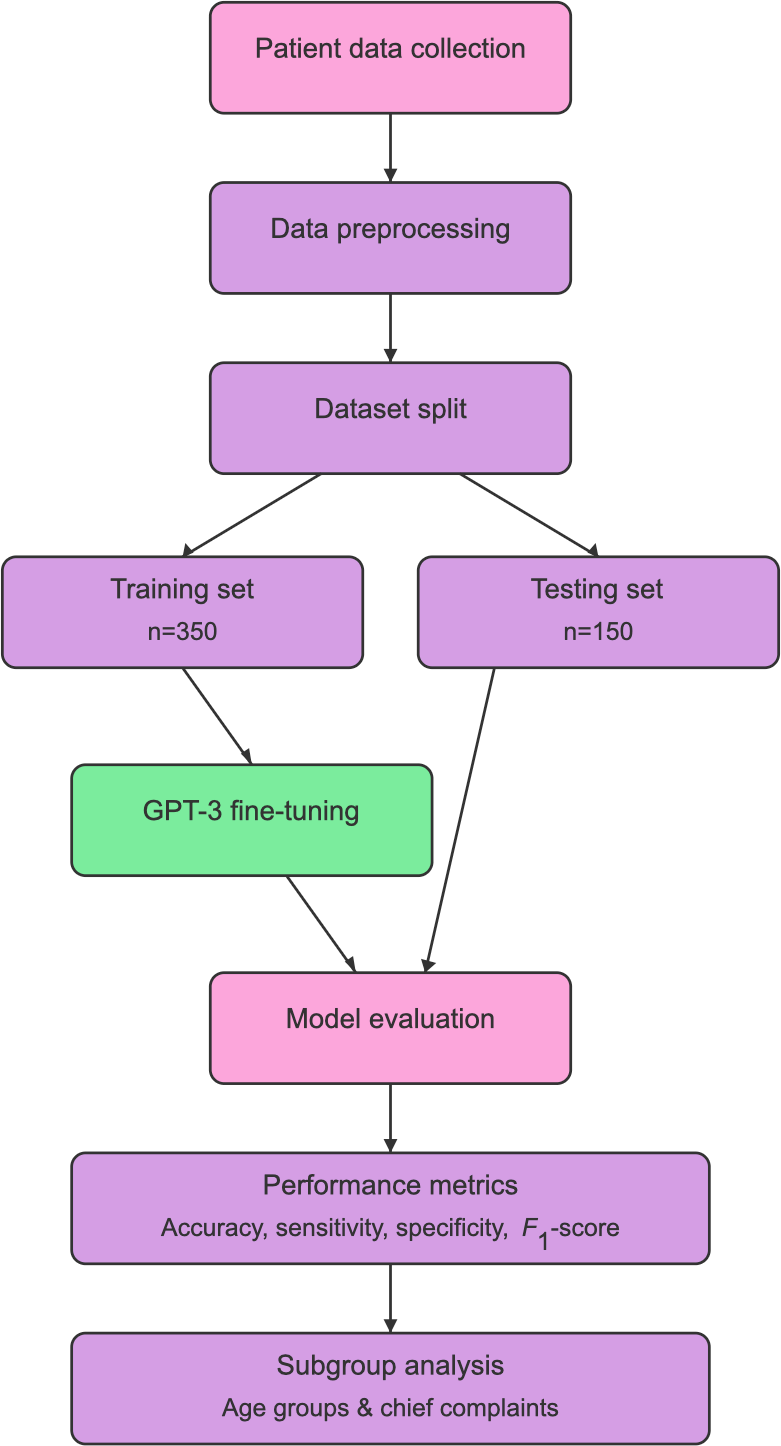
Workflow schematic showing the process of data collection, preprocessing, model training, and evaluation. The pipeline includes data splitting (70% training, 30% testing), GPT-3 fine-tuning, and comprehensive performance evaluation including subgroup analyses.

This multicenter retrospective cohort study was conducted in collaboration with a rural pediatric health care organization in Central Louisiana. The organization provides primary care to approximately 15,000 pediatric patients. The study analyzed patient data collected between January 2020 and December 2021. The overall workflow of the study is illustrated in Figure 1, encompassing data collection through model evaluation.

### Ethical Considerations

Ethics approval was obtained from the Mansoor Pediatrics Ethics Committee (approval MP-2023‐017), and the study adhered to the principles of the Declaration of Helsinki. The study used retrospective, deidentified patient data and was exempt from informed consent requirements. Data were anonymized to ensure compliance with Health Insurance Portability and Accountability Act (HIPAA) regulations. No identifying information was accessible to researchers. No compensation was provided to participants as the study relied on existing retrospective data. For secondary analyses using deidentified data, the original consent obtained at the time of patient care covered the use of the data for research purposes.

### Participants and Data Collection

A total of 500 pediatric patient encounters were included based on the following criteria:

- Inclusion criteria: Patients aged 0‐18 years with a documented chief complaint and pediatrician-generated differential diagnosis
- Exclusion criteria: Encounters with incomplete or inconsistent data

Anonymized data, including patient age, sex, chief complaint, presenting symptoms, medical history, and pediatrician-generated differential diagnoses, were extracted from the EHR system. Two independent researchers manually reviewed the data to ensure accuracy and consistency. No missing data were present in the final dataset. Demographic information, including racial and ethnic background, was not collected as part of this dataset. This omission limits the ability to assess potential biases in model performance across racial or ethnic groups, which is an important consideration for future research.

Five board-certified pediatricians (mean experience: 12, SD 5.8, range 5‐20 years) participated in the study as reference standard providers. Pediatricians were recruited from the participating health care organization based on their availability and experience in rural pediatrics.

### Data Preprocessing

For each patient encounter, the chief complaint, presenting symptoms, and relevant medical history were concatenated into a single text string. Identifying information was removed to ensure privacy. Medical terms were standardized using a medical dictionary, and data were formatted for compatibility with the GPT-3 model.

### Model Training and Fine-Tuning

The GPT-3 model (DaVinci version) was fine-tuned using the OpenAI application programming interface. The dataset was randomly split into a training set (n=350, 70%) and a testing set (n=150, 30%). The model was trained to generate up to five differential diagnoses for each input case. The study used retrospective data that included pediatrician-generated differential diagnoses documented during actual clinical encounters. No pediatricians were prospectively instructed to generate differential diagnoses specifically for this study. The same format of up to 5 differential diagnoses was used for standardization when processing both the historical physician documentation and the GPT-3 outputs. Fine-tuning parameters included 10 epochs, a batch size of 4, and a learning rate of 1e-5. The fine-tuning process aimed to optimize the model’s ability to generate accurate and relevant differential diagnoses based on the input data. These details are visible in Multimedia Appendix 1.

GPT-3 (DaVinci version) was selected for this study because it was the most advanced version of the GPT model available at the time of data collection and model fine-tuning. Subsequent versions, such as GPT-3.5 and GPT-4, were released after the study period and were therefore not considered. Future work could explore the performance of these newer models in similar settings to assess potential improvements in diagnostic accuracy.

### Evaluation Metrics

The model’s performance was evaluated using the following metrics (Table 1):

- Accuracy: Proportion of correct predictions (true positives and true negatives) relative to total cases
- Sensitivity (recall): Proportion of actual positive diagnoses correctly identified by the model
- Specificity: Proportion of actual negative diagnoses correctly excluded by the model
- Precision: Proportion of positive predictions that were correct
- *F*_1_-score: Harmonic mean of precision and sensitivity

In addition to these metrics, subgroup analyses were conducted by age group (0‐5, 6‐12, and 13‐18 years) and chief complaints (eg, fever, abdominal pain).

**Table 1.** Testing set evaluation metrics for analysis of the fine-tuned GPT-3 model, including formulas and values of the evaluation metrics for the GPT-3 model.

Metric	Formula	Description
Sensitivity (recall)	TP^a^^,^^b^/(TP + FN^c^^,^^d^)	The proportion of actual positive diagnoses that were correctly identified by the model
Specificity	TN^e^^,^^f^/(TN + FP^g^^,^^h^) 0.90	The proportion of actual negative diagnoses that were correctly identified by the model
Precision	TP/(TP + FP)	The proportion of the model’s positive predictions that were actual positive diagnoses
*F*_1_-score	2 * (precision * sensitivity)/(precision + sensitivity)	The harmonic mean of precision and sensitivity, providing a balanced measure of the model’s performance
Accuracy	(TP + TN)/(TP + TN + FP + FN)	The overall proportion of correct predictions made by the model

a TP: true positive.

b Cases where the model correctly predicted a positive diagnosis.

c FN: false negative.

d Cases where the model incorrectly predicted a negative diagnosis.

e TN: true negative.

f Cases where the model correctly predicted a negative diagnosis.

g FP: false positive.

h Cases where the model incorrectly predicted a positive diagnosis.

### Statistical Analysis

Descriptive statistics were used to summarize patient demographics and model performance. *χ*^2^ tests were used for categorical variables, and independent 2-tailed *t* tests were used for continuous variables. Statistical significance was set at *P*<.05. Data normality was assessed using the Kolmogorov-Smirnov test before statistical analysis. Our outcome metrics (accuracy, sensitivity, specificity) were found to follow a normal distribution (*P*>.05), supporting our use of parametric statistical methods including *t* tests for comparisons between groups. For nonnormally distributed variables, nonparametric alternatives (Mann-Whitney *U* test) were applied.

*χ*^2^ tests were chosen for categorical variables due to their robustness in comparing proportions across groups. Independent *t* tests were selected for continuous variables after confirming normality of distribution. The choice of metrics (accuracy, sensitivity, specificity) aligns with standard diagnostic evaluation frameworks in health care AI validation studies. Subgroup analyses were performed to assess model performance consistency across demographics and clinical presentations, which is essential for evaluating potential biases in model predictions.

Power analysis indicated that a sample size of 500 would provide 80% power to detect a 10% difference in accuracy between the GPT-3 model and pediatricians, assuming a pediatrician accuracy of 90%. This calculation accounted for the expected distribution of common and rare diagnoses in our pediatric population, with consideration for potential subgroup analyses across different age groups and chief complaints.

### Software and Tools

The statistical analysis was conducted using Python 3.8 (Python Software Foundation) [19] with the scikit-learn library [20] for model evaluation and SPSS Statistics version 29 (IBM Corp) for additional analysis [21]. The OpenAI application programming interface was used for model fine-tuning and prediction generation [22]. Software and scripts used in this study are available upon request for reproducibility.

## Results

### Dataset Characteristics

A total of 500 pediatric patient encounters were included, with 350 (70%) cases in the training set and 150 (30%) cases in the testing set. The mean age of patients was 7.5 (SD 5.2) years, and 52.2% (n=261) of participants were female. The most common chief complaints were fever (n=130, 26%), cough (n=98, 19.6%), abdominal pain (n=73, 14.6%), and rash (n=49, 9.8%). The distribution of age, sex, and chief complaint was similar between the training and testing sets (Table 2).

**Table 2.** Demographics and dataset characteristics.

Characteristic	Total (N=500)	Training set (n=350)	Testing set (n=150)	*P* value
Age (years), mean (SD)	7.5 (5.2)	7.4 (5.1)	7.7 (5.3)	.56^a^
Sex, n (%)				.82^b^
Female	261 (52.2)	184 (52.6)	77 (51.3)	
Male	239 (47.8)	166 (47.4)	73 (48.7)	
Chief complaint, n (%)				.93^b^
Fever	130 (26.0)	91 (26.0)	39 (26.0)	
Cough	98 (19.6)	70 (20.0)	28 (18.7)	
Abdominal pain	73 (14.6)	50 (14.3)	23 (15.3)	
Rash	49 (9.8)	34 (9.7)	15 (10.0)	
Other	150 (30.0)	105 (30.0)	45 (30.0)	
Rare diagnoses, n (%)	20 (4.0)	14 (4.0)	6 (4.0)	>.99

a *P* value calculated using independent 2-tailed *t* test.

b *P* value calculated using *χ*2 test.

### Model Performance

The fine-tuned GPT-3 model achieved high accuracy in generating differential diagnoses on the testing set. Key performance metrics are as follows:

- Accuracy: 87.3% (131/150 cases)
- Sensitivity (recall): 85% (95% CI 82%‐88%)
- Specificity: 90% (95% CI 87%‐93%)
- Precision: 89% (95% CI 86%‐92%)
- *F*_1_-score: 0.87

The model correctly identified 128 positive diagnoses and excluded 334 negative diagnoses, with 16 false positives and 22 false negatives.

### Subgroup Analysis

Performance across age groups and common chief complaints are summarized in Tables2  3. The model’s accuracy was consistent across age groups:

- 0‐5 years: 87% (54/62 cases)
- 6‐12 years: 89% (47/53 cases)
- 13‐18 years: 86% (30/35 cases)

**Table 3.** Model performance by common chief complaints.

Chief complaint	Accuracy (95% CI)	Sensitivity (95% CI)	Specificity (95% CI)	Precision (95% CI)	*F*_1_-score (95% CI)
Fever (n=39)	0.92 (0.88-0.96)	0.90 (0.85-0.95)	0.93 (0.90-0.96)	0.92 (0.87-0.97)	0.91 (0.86-0.96)
Cough (n=28)	0.89 (0.82-0.94)	0.85 (0.79-0.91)	0.90 (0.84-0.92)	0.89 (0.83-0.95)	0.87 (0.81-0.93)
Abdominal pain (n=23)	0.87 (0.78-0.92)	0.82 (0.75-0.89)	0.87 (0.83-0.90)	0.86 (0.79-0.93)	0.84 (0.77-0.91)
Rash (n=15)	0.93 (0.83-0.97)	0.88 (0.80-0.96)	0.91(0.88-0.94)	0.90 (0.92-0.98)	0.89 (81-0.97)

Similarly, the model demonstrated robust performance for common chief complaints:

- Fever: 92% (36/39 cases) accuracy
- Cough: 89% (25/28) accuracy
- Abdominal pain: 87% (20/23) accuracy
- Rash: 93% (14/15) accuracy

Subgroup analyses by age group and chief complaints revealed consistent performance, indicating the model’s ability to generalize across varying pediatric presentations. However, the slight performance drop in complex and rare cases underscores the importance of targeted training datasets for improving diagnostic accuracy in these subgroups. For rare or complex diagnoses (n=20), the model achieved an accuracy of 80% (16/20 cases), slightly lower than the overall accuracy but comparable to pediatricians (17/20, 85% of cases; *P*=.62).

### Comparison With Pediatricians

The model’s performance was comparable to that of the 5 participating board-certified pediatricians. Pediatricians achieved an accuracy of 91.3% (137/150 cases), with a sensitivity of 92% (95% CI 91%-94%) and specificity of 88% (95% CI 84%-90%). Differences in sensitivity (*P*=.08) and specificity (*P*=.57) between the model and pediatricians were not statistically significant.

### Statistical Analysis

*χ*^2^ tests indicated no significant differences between the GPT-3 model and pediatricians for accuracy, sensitivity, or specificity. Subgroup analyses confirmed consistent performance across age groups and common chief complaints, with no significant performance disparities.

### Tables

Table 1 provides a detailed breakdown of the evaluation metrics. Table 4 shows the performance of the model by age group, while Table 3 summarizes performance by chief complaints.

**Table 4.** Model performance by age group.

Age group (years)	Accuracy (95% CI)	Sensitivity (95% CI)	Specificity (95% CI)	Precision (95% CI)	*F*_1_-score (95% CI)
Overall (n=150)	0.85 (0.81-0.89)	0.90 (0.87-0.93)	0.89 (0.86-0.92)	0.87 (0.83-0.91)	0.88 (0.85-0.91)
0‐5 (n=62)	0.87 (0.82-0.92)	0.84 (0.79-0.89)	0.89 (0.85-0.93)	0.88 (0.83-0.93)	0.86 (0.81-0.91)
6‐12 (n=53)	0.89 (0.84-0.94)	0.86 (0.81-0.91)	0.91 (0.87-0.95)	0.90 (0.85-0.95)	0.88 (0.83-0.93)
13‐18 (n=35)	0.86 (0.80-0.92)	0.83 (0.77-0.89)	0.88 (0.83-0.93)	0.87 (0.81-0.93)	0.85 (0.79-0.91)

## Discussion

### Principal Findings

This study evaluated the diagnostic performance of a fine-tuned GPT-3 model in generating pediatric differential diagnoses in rural health care settings. The model achieved an accuracy of 87%, which was comparable to board-certified pediatricians’ accuracy of 91%. Performance was consistent across age groups and common chief complaints, underscoring the model’s potential as a reliable clinical decision support tool. While the model demonstrated lower accuracy for rare or complex cases (80%), its performance remained comparable to that of pediatricians (85%). These findings suggest that LLMs could enhance diagnostic accuracy and support providers in underserved regions, particularly for routine presentations.

### Comparison to Prior Work

Our findings align with prior studies demonstrating the potential of LLMs in clinical decision support. For example, Steinberg et al [7] reported 82% accuracy in adult diagnostic support using LLMs, while Wu et al [11] achieved 97.45% accuracy in pediatric otitis media interpretation with deep learning models. This study extends these findings by focusing on general pediatric differential diagnosis, an area with limited prior research. Unlike previous studies that primarily examined urban or hospital-based datasets, our work highlights the utility of LLMs in resource-constrained rural environments, addressing a critical gap in the literature.

### Strengths and Limitations

This study has several strengths. First, the use of real-world data from rural health care settings enhances the generalizability of findings to similar environments. Second, the inclusion of subgroup analyses provides insights into the model’s performance across diverse age groups and chief complaints. Third, the comparative evaluation with experienced pediatricians underscores the model’s clinical relevance.

Another of the key strengths of this study lies in its real-world applicability, particularly for rural health care settings where resources are limited and access to specialists is often constrained. By leveraging existing EHR data and evaluating the model’s performance on common and rare pediatric conditions, this research provides a practical framework for integrating AI tools into primary care workflows. The consistent accuracy demonstrated across age groups and chief complaints highlights the potential of GPT-3 to serve as a valuable diagnostic support system for providers in underserved areas. However, implementing such tools in real-world clinical settings will require addressing infrastructure challenges, including internet connectivity and provider training. Despite these challenges, the findings underscore the feasibility of deploying AI systems to enhance diagnostic accuracy and reduce disparities in health care delivery, particularly in environments with high patient volumes and limited specialist availability.

However, there are notable limitations:

- Sample size and diversity: The sample size of 500 encounters, while informative, may not fully capture the diversity of the broader pediatric population. This limitation is particularly relevant in diverse health care settings, where factors such as demographic variability, socioeconomic status, and health care access can influence diagnostic patterns. Prior studies have demonstrated that models trained on limited datasets often fail to generalize across different populations, highlighting the need for larger, multi-institutional datasets to improve validity and applicability [17]. Additionally, our study used data from a single rural health care organization, which may limit the external validity of our findings. Similar studies have shown that AI-based diagnostic models exhibit performance degradation when applied to new patient populations due to variations in disease prevalence, clinical workflows, and physician documentation styles [18]. For instance, Steinberg et al [7] found that an LLM trained on one hospital’s EHRs experienced a 15% drop in accuracy when tested on data from a different institution. These findings emphasize the need for external validation. Future research should prioritize expanding the sample size through multicenter collaborations, incorporating data from health care centers with diverse patient demographics to enhance generalizability and robustness. Similar initiatives have demonstrated improved AI model performance when trained on heterogeneous datasets, such as the multi-institutional validation study by Rajkomar et al [2], which improved diagnostic accuracy across multiple health care networks.
- Retrospective design: The use of retrospective data limits the ability to assess the model’s impact on clinical workflows or patient outcomes. Prospective clinical trials are needed to evaluate these aspects.
- Cross-validation: A key limitation of this study is the lack of cross-validation across different health care organizations. Evidence suggests that AI-based diagnostic models frequently underperform when tested on external datasets due to variations in clinical documentation, patient demographics, and institutional practices. For example, a systematic review of AI applications in health care found that models trained on single-center data exhibited an average 12%‐20% decrease in performance when applied to external datasets [17]. Steinberg et al [7] also demonstrated that LLMs trained on EHRs from one hospital struggled to maintain accuracy when exposed to unseen patient populations, emphasizing the importance of cross-validation. Furthermore, ChatGPT-based diagnostic models have shown variability in reliability across different patient demographics, particularly when applied to pediatric populations with rare conditions [12]. To ensure reproducibility, future studies should incorporate external validation using data from multiple institutions, including urban, suburban, and rural health care settings. By validating performance across diverse patient populations, we can assess the model’s reliability in real-world clinical environments and mitigate the risks associated with dataset bias. This approach aligns with recommendations from previous research advocating for multicenter validation to improve AI model robustness [18].
- Rare diagnoses: The model’s lower accuracy for rare or complex cases highlights the need for further fine-tuning and testing in these areas. Future fine-tuning efforts could incorporate domain-specific datasets, such as rare pediatric conditions or uncommon presentations, to enhance the model’s diagnostic accuracy for less frequently encountered cases. For example, fine-tuning could focus on rare pediatric conditions such as Kawasaki disease or metabolic disorders, which often present atypically and are prone to diagnostic errors. Collaborations with specialist clinics could help build robust datasets for such conditions.
- GPT-3 versus newer models: Another limitation is the use of GPT-3 instead of its newer iterations, such as GPT-3.5 or GPT-4, which were released after the completion of this study. While GPT-3 demonstrated strong diagnostic performance, future studies should evaluate whether these more advanced models can further enhance accuracy, particularly for rare or complex cases. Specifically, GPT-3.5 and GPT-4 feature enhanced contextual understanding and larger training corpora [23], which may improve their ability to identify nuanced patterns in rare pediatric diagnoses. Additionally, these models may mitigate hallucination risks and offer better attribution of sources, which are critical for clinical applications. Comparative evaluations in similar rural health care settings would provide insights into their incremental benefits over GPT-3.

### Practical Implications

Integrating LLMs like GPT-3 into rural health care settings could address critical challenges such as physician shortages, high patient volumes, and limited specialist access. These tools can provide rapid accurate diagnostic support, reducing diagnostic errors and improving patient outcomes [24]. However, practical barriers to implementation, including infrastructure requirements (eg, reliable internet and electricity) and provider training, must be addressed [25].

Reliance on AI systems poses risks, including overreliance by less experienced providers and challenges in managing incomplete or inconsistent input data [26]. Training programs should ensure health care providers understand the limitations of AI tools and develop strategies for validating AI-generated outputs. Establishing clear guidelines for AI use in clinical settings will further ensure patient safety and ethical application. To address concerns about hallucinations—instances where the model generates inaccurate or fabricated information—health care providers must verify AI-generated outputs against clinical guidelines and existing evidence. Integrating feedback mechanisms, where physicians can flag inaccuracies, may also help refine model behavior over time [27].

Additionally, fostering trust in AI tools among providers and patients will be essential for successful adoption [28]. Additionally, parental concerns regarding deferring diagnostic decisions to AI systems must be addressed to build trust and acceptance. Efforts to educate families about AI’s role as a supplementary decision-making tool rather than a replacement for physician judgment are essential. Furthermore, rural health care facilities may face challenges in implementing AI solutions due to limited infrastructure, such as inconsistent internet access, power supply, and provider training [29]. These challenges may also include the cost of deploying and maintaining AI systems, as well as the need for ongoing technical support. Policy makers and health care administrators should explore subsidized programs or partnerships with technology providers to ensure equitable access to AI tools in resource-limited settings. Addressing these barriers will be crucial for ensuring successful adoption and integration into clinical workflows.

### Future Directions

Future research should focus on the following:

- The findings should be validated in larger, more diverse populations across multiple health care settings.
- The diagnostic capabilities of more advanced models, such as GPT-3.5 or GPT-4, should be assessed to determine whether recent improvements in language model architecture further enhance diagnostic accuracy.
- The impact of LLM integration on patient outcomes, provider satisfaction, and workflow efficiency in prospective clinical trials should be assessed.
- User-friendly interfaces should be developed to facilitate adoption by providers with varying levels of technological expertise, and training programs tailored to rural health care providers should be developed to familiarize them with AI tools and address potential apprehensions about using such systems. These programs should emphasize the complementary nature of AI in clinical workflows rather than its replacement of human judgment.
- Ethical concerns, including data privacy, informed consent, and model transparency, should be addressed to ensure responsible use in clinical practice.
- In addition to traditional evaluation metrics, future studies should assess language generation issues such as hallucinations—instances where the model produces false or unsupported information—and attribution of responses to reliable sources.

These factors are critical for ensuring the safety and reliability of AI applications in clinical decision-making. Natural language processing metrics like Recall-Oriented Understudy for Gisting Evaluation (ROUGE) and bilingual evaluation understudy (BLEU) may be used to evaluate output quality, while further human review of generated responses could assess alignment with established clinical guidelines.

### Conclusions

This study highlights the potential of GPT-3, a fine-tuned LLM, as a clinical decision support tool for pediatric differential diagnosis in rural health care settings. The model achieved diagnostic accuracy comparable to that of board-certified pediatricians, demonstrating robust performance across age groups and common presenting complaints. These findings suggest that LLMs could serve as valuable tools for addressing the unique challenges faced by rural health care providers, such as limited access to specialists and high patient volumes.

However, this work also underscores the need for further validation. Future research should focus on evaluating the model’s performance in larger, diverse populations and real-world clinical settings. Ethical considerations, including data privacy and model transparency, must be prioritized to ensure responsible implementation. Another ethical consideration is the potential for AI models to exacerbate existing health disparities if their development does not account for diverse populations. Rigorous testing in underrepresented groups and ongoing audits for bias are critical steps to ensure fairness and equity in AI-driven health care applications. By addressing these challenges, LLMs like GPT-3 have the potential to enhance diagnostic accuracy, reduce disparities in access to care, and improve outcomes for pediatric patients in underserved regions.

While this study represents a step toward integrating AI into rural health care, its findings underscore the need for iterative improvements and cross-disciplinary collaboration to refine these tools. Partnerships between AI developers, clinicians, and health care administrators will be crucial in ensuring that AI solutions are both effective and accessible.

This study serves as a step in bridging the gap between AI innovation and practical health care applications, paving the way for future advancements in clinical decision support systems tailored to the needs of rural health care environments.

## Supplementary material

10.2196/65263 Multimedia Appendix 1 Technical appendix: GPT-3 Model specifications and implementation details.

## Abbreviations

AI: artificial intelligence
BLEU: bilingual evaluation understudy
EHR: electronic health record
HIPAA: Health Insurance Portability and Accountability Act
LLM: large language model
ROUGE: Recall-Oriented Understudy for Gisting Evaluation
