# Supplementary material for: Large Language Models for Pediatric Differential Diagnoses in Rural Health Care: Multicenter Retrospective Cohort Study Comparing GPT-3 With Pediatrician Performance
Source: JMIRx Med. 2025 Mar 19;6:e65263. doi: 10.2196/65263 (PMC11939124; doi:10.2196/65263)
Supplement: Multimedia Appendix 1 [file xmed-v6-e65263-s001.docx]

Technical Appendix: GPT-3 Model Specifications and Implementation Details

1. GPT-3 Model Architecture Specifications

This study utilized the GPT-3 DaVinci model, which is based on a transformer architecture with the following specifications:

- **Model Version**: GPT-3 (davinci)
- **Parameters**: 175 billion parameters
- **Architecture**: Decoder-only transformer network
- **Attention Heads**: 96 attention heads
- **Layers**: 96 transformer layers
- **Context Window**: 4,096 tokens
- **Training Corpus**: 570GB of text data from diverse internet sources, books, and articles
- **Embedding Dimensions**: 12,288 dimensions

The model's multi-head attention mechanism enables it to capture complex relationships in text, making it suitable for medical reasoning tasks such as differential diagnosis generation.

2. Fine-Tuning Methodology

*2.1 Pre-processing and Tokenization*

- **Tokenizer**: GPT-3's byte-pair encoding (BPE) tokenizer was used
- **Data Preprocessing**:
  - Standardization of medical terminology
  - Removal of identifying information
  - Concatenation of chief complaint, symptoms, and relevant history
  - Uniform formatting of input text

*2.2 Fine-Tuning Hyperparameters*

The model was fine-tuned using the OpenAI API with the following hyperparameters:

- **Learning Rate**: 1e-5 with cosine decay schedule
- **Batch Size**: 4
- **Epochs**: 10
- **Optimizer**: Adam with β1 = 0.9, β2 = 0.999, ε = 1e-8
- **Weight Decay**: 0.1
- **Gradient Accumulation Steps**: 4
- **Warmup Steps**: 500 (10% of total steps)
- **Maximum Sequence Length**: 1,024 tokens
- **Dropout Rate**: 0.1 for regularization

*2.3 Training Process*

- **Hardware**: Fine-tuning performed on OpenAI's servers via API
- **Training Time**: Approximately 4 hours
- **Early Stopping**: Implemented with patience of 3 epochs
- **Loss Function**: Cross-entropy loss
- **Validation Split**: 10% of training data reserved for validation during fine-tuning
- **Checkpoint Selection**: Best performing checkpoint based on validation loss

3. Token Limitations and Input Processing

The model operated within the following token constraints:

- **Maximum Input Length**: 1,024 tokens
- **Maximum Output Length**: 256 tokens
- **Token Handling Strategy**: For cases where input exceeded token limits, we implemented a summarization strategy that preserved key clinical information, prioritizing:
  - Primary complaint
  - Current symptoms
  - Vital signs
  - Relevant medical history

4. Prompt Engineering Methodology

*4.1 Prompt Structure*

We developed a consistent prompt template to ensure reliable model performance:

Patient Information:

Age: [PATIENT_AGE] years

Gender: [PATIENT_GENDER]

Chief Complaint: [CHIEF_COMPLAINT]

Presenting Symptoms: [SYMPTOMS]

Relevant Medical History: [MEDICAL_HISTORY]

Based on the above information, provide a ranked list of the top 5 most likely differential diagnoses for this pediatric patient.

*4.2 Prompt Refinement Process*

The prompt structure underwent iterative refinement during development:

1. Initial testing with 50 cases to assess prompt effectiveness
2. Analysis of model errors and pattern identification
3. Prompt adjustments to improve accuracy
4. Validation on a separate set of 25 cases
5. Final prompt selection based on performance metrics

5. Validation Approach

*5.1 Evaluation Protocol*

- **Cross-validation**: 5-fold cross-validation was performed within the training set
- **Test Set Isolation**: The test set (30% of data) was completely isolated from the training process
- **Stratification**: Test set selection was stratified by age group and chief complaint to ensure representative distribution
- **Evaluator Blinding**: The five pediatricians who evaluated model outputs were blinded to whether a differential diagnosis was generated by the model or by another physician

*5.2 Quality Control Measures*

- **Inter-rater Reliability**: Cohen's kappa coefficient between physician evaluators was 0.83, indicating strong agreement
- **Consistency Checking**: 10% of cases were randomly selected for double evaluation to assess consistency
- **Error Analysis**: Qualitative analysis of false positives and false negatives to identify systematic errors
- **Confidence Calibration**: The model's confidence scores were calibrated using Platt scaling

6. Output Processing

*6.1 Post-processing Algorithm*

Model outputs underwent systematic post-processing:

1. Extraction of explicitly mentioned diagnoses
2. Standardization of terminology using SNOMED-CT mappings
3. Removal of duplicates and consolidation of related diagnoses
4. Ranking preservation from the model's output
5. Verification that outputs contained exactly 5 diagnoses (or fewer if the model provided fewer)

*6.2 Handling of Rare Conditions*

For rare conditions, additional verification steps were implemented:

1. Cross-reference with pediatric databases for clinical plausibility
2. Verification that presenting symptoms aligned with proposed rare diagnoses
3. Consultation with specialist pediatricians for ambiguous cases

7. Reproducibility Information

- **Random Seed**: 42 (used for reproducibility)
- **Software Versions**:
  - Python 3.8.12
  - OpenAI API (version used during study period: 2023-05)
  - scikit-learn 1.0.2
  - pandas 1.3.5
  - numpy 1.21.5
  - SPSS Statistics 28.0

8. Limitations of the Technical Approach

- **Black Box Nature**: The internal reasoning process of GPT-3 remains largely opaque
- **Token Limitations**: Complex cases may hit token limits, requiring summarization
- **Temporal Knowledge Cutoff**: GPT-3's knowledge cutoff date preceded some recent medical advances
- **Hallucination Risk**: GPT-3 can potentially generate plausible-sounding but incorrect information
- **Parameter Access Limitations**: Full access to model parameters was limited by API constraints

9. Model Safety and Bias Mitigation

- **Safety Filters**: OpenAI's content filtering systems were active during model deployment
- **Bias Testing**: We assessed the model for gender and age biases across different demographic groups
- **Uncertainty Communication**: The model was configured to express uncertainty when appropriate rather than making high-confidence incorrect predictions
- **Human-in-the-Loop Design**: The system was designed with the assumption that outputs would be reviewed by healthcare professionals
